# A very rare presentation of lung cancer: Metastases to the distal phalanx of index–case report

**DOI:** 10.1097/MD.0000000000017892

**Published:** 2019-12-10

**Authors:** Vlad-Adrian Afrăsânie, Anca Maria Adavidoaiei, Iuliana Hunea Zamisnicu, Ionut Gabriel Funingănă, Mihai Vasile Marinca, Bogdan Gafton, Dana Elena Clement, Marius-Ionut Păduraru, Irina Demşa, Lucian Miron, Teodora Alexa-Stratulat

**Affiliations:** a “Gr.T. Popa” University of Medicine and Pharmacy; b Medical Oncology Department, Regional Institute of Oncology; c Cardiology Department, Emergency Hospital “Sf. Spiridon,” Iaşi, Romania; d Early Phase Clinical Trials Unit, Addenbrooke's Hospital, Cambridge, UK.

**Keywords:** acrometastases, case report, index finger, lung adenocarcinoma

## Abstract

**Rationale:**

Acrometastases of the hand are an unusual sign of lung cancer onset and may often be mistaken for other benign disorders, thus delaying diagnosis and treatment.

**Patient concerns:**

A 58-year-old man presented at the Rheumatology Clinic with a lump in the distal phalanx of the right index finger associated with intense pain, swelling, rib pain, and hemoptysis.

**Diagnoses:**

Given the clinical manifestations, an x-ray of the right hand was performed, and it revealed an osteolytic lesion in the distal phalanx of the right index finger. The subsequent CT of the thorax and abdomen showed a lung tumor, osteolytic lesions in the ribs, sternum, and the thoracic spine.

**Interventions:**

Amputation of the phalanx was decided on account of intense pain refractory to NSAIDs and opioids. Pathology assessment established the diagnosis of bone metastases secondary to lung adenocarcinoma. The patient underwent 6 cycles of first-line palliative chemotherapy with cisplatin and gemcitabine with partial response according to the RECIST 1.1. criteria. EGFR and ALK testing were not available at the time. A year later, the patient presented with progressive disease, which lead to 6 more cycles of chemotherapy with docetaxel. The disease progressed during chemotherapy and the patient was switched to erlotinib.

**Outcomes:**

After 7 months of anti-EGFR treatment, the patient passed away due to disease progression, thus having an overall survival of 25 months.

**Lessons:**

On rare occasions, acrometastases of the hand may be the first manifestation of a lung cancer and, as such, they must be taken into consideration in the differential diagnosis of rheumatologic disorders. They are a poor prognosis marker, but some cases like this one can have a better survival than reported in the literature, most likely due to that particular cancer's biology.

## 1 Introduction

Lung cancer is one of the most aggressive types of tumors in human pathology and accounts for the greatest number of cancer-related deaths worldwide.^[1]^ Most cases are diagnosed in their metastatic stage.^[2]^ The metastatic patterns may be very different, and localizations of secondary lesions in almost every organ are described in the literature. Most often, metastatic sites in lung cancer are the lungs, the liver, and the bone.^[2]^ Secondary bone lesions are most commonly located in the vertebrae, pelvis, ribs, and sternum.^[1]^ Rarely, they may occur in the bones of the hand or foot, in which case they are named acrometastases.^[3]^ Lung cancer is the main neoplasm causing acrometastases in the hand, followed by kidney and breast cancer.^[4]^ The literature describes 24 such cases.^[5]^ The lesions are most frequently situated in the thumb and least so in the carpal area.^[4]^ Acrometastases very rarely represent the first signs and symptoms of stage IV lung neoplasm and this tends to delay diagnosis; the differential diagnosis of a tumor in the fingers is complex and includes arthritis, osteomyelitis, trauma, gout, Paget disease, cysts, benign, or malignant tumors of the bones or skin. The acrometastases of lung cancer are associated with poor prognosis. The average survival is ∼6 to 7 months, compared to that of 9.7 months in the case of stage IV patients.^[2,6–8]^

We are hereby reporting the case of a patient with acrometastases in the distal phalanx of the right index finger, secondary to lung adenocarcinoma. Although the treatment of acrometastases is not standardized, the therapeutic results in this case were favorable and led to extended survival and a good quality of life to this patient, also facilitated by early diagnosis. The patient's survival was 4 times the average rate reported in the literature, which leads us to question how acrometastases impact prognosis.

## 2 Case presentation

**Figure 1:**
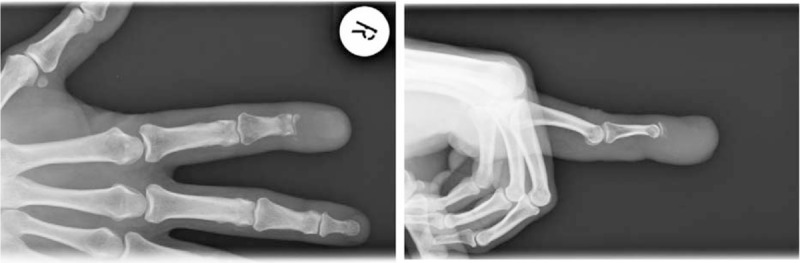
Hand X-ray: PA and lateral view.

**Figure 2:**
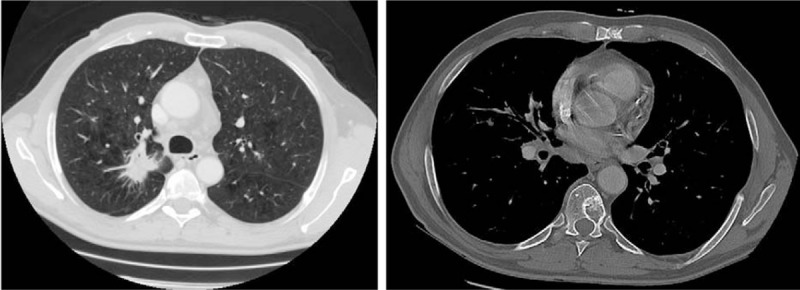
Lung tumor located in the right upper lobe and thoracic lumbar metastases.

**Figure 3:**
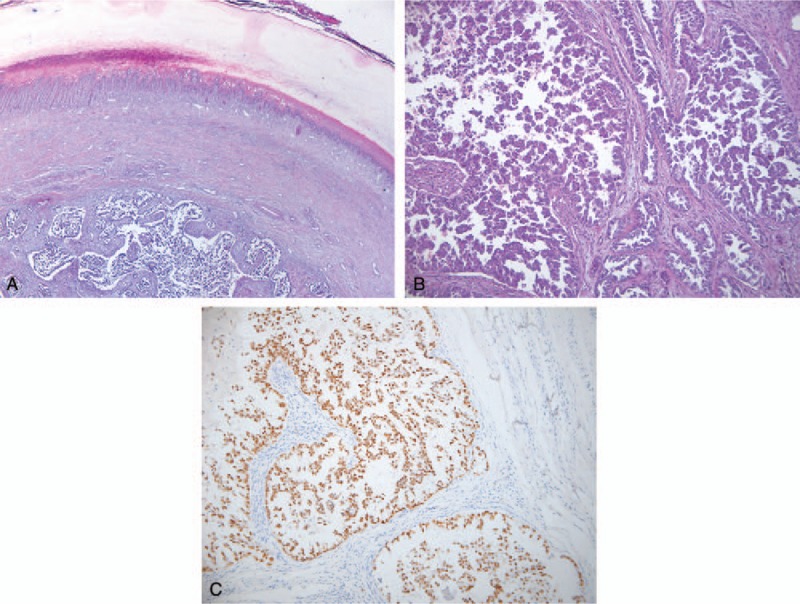
Histopathological examination of the acrometastases: (A) adenocarcinoma infiltration in the nail bed (H&E), (B) tumor proliferation with acinar, tubular, and micropapilary pattern (H&E, ×100), (C) TTF1 ×100 diffusely positive (nuclear markers).

A Caucasian, 58-year-old retired Lieutenant presented at the Rheumatology Clinic in February 2013 complaining of an intensely painful lump in the distal phalanx of the right index finger, not responding to NSAIDs and opioids, associated with swelling rib pain and hemoptysis. Anamnesis did not reveal any personal, hereditary, or collateral history of cancer. Noteworthy from the medical history was a diagnosis of grade 3 essential hypertension kept under control by ongoing treatment with 40 mg/day of telmisartan and 10 mg/day of amlodipine. Also, the patient was a smoker of 30 pack-years. The physical exam revealed decreased pulmonary sounds in the right upper thorax, and 135/85 mm Hg arterial pressure. The patient also had grade 1 anemia – Hb-13 g/dL and a slightly increased LDH value of 244 U/L. Given the symptoms, an x-ray of the right hand was performed, revealing cvasicomplete distal phalanx osteolysis in the index finger (Fig. 1). This led to the suspicion of neoplasm and, taking into consideration the other symptoms, a CT scan of the thorax and abdomen was recommended. The CT showed a 30/28/17 mm tumor in the posterior segment of the right upper lobe (Fig. 2), infiltrated intrathoracic lymph nodes and other adenopathies: 8/15 mm right inferior paratracheal, 16/10 mm in the aortopulmonary window, 11/6 mm paraaortic, 28/14 mm subcarinal, as well as osteolytic bone lesions located in the sternum, T7 and T8 vertebrae (Fig. 2), C1 and C9 right costal arches, and C3, C4, C5, C7, C8, and C12 left costal arches. In order to confirm the diagnosis, the patient was referred to the Plastic Surgery Clinic, where he underwent the amputation of the distal phalanx. The macroscopic histopathologic exam showed complete osteolysis of the distal segment of the phalanx. The microscopic analysis revealed a well-differentiated adenocarcinoma bone metastase in the distal phalanx, with papillary and micropapillary architecture and complete osteolysis (Fig. 3A and B). Immunohistochemical staining tests confirmed a cytokeratin 7 positive tumor and the TTF-1 was diffusely positive (Fig. 3C). The diagnosis of stage IV lung adenocarcinoma cT2aN3M1b was established in accordance with the 7th edition of AJCC staging.

**Figure 4:**
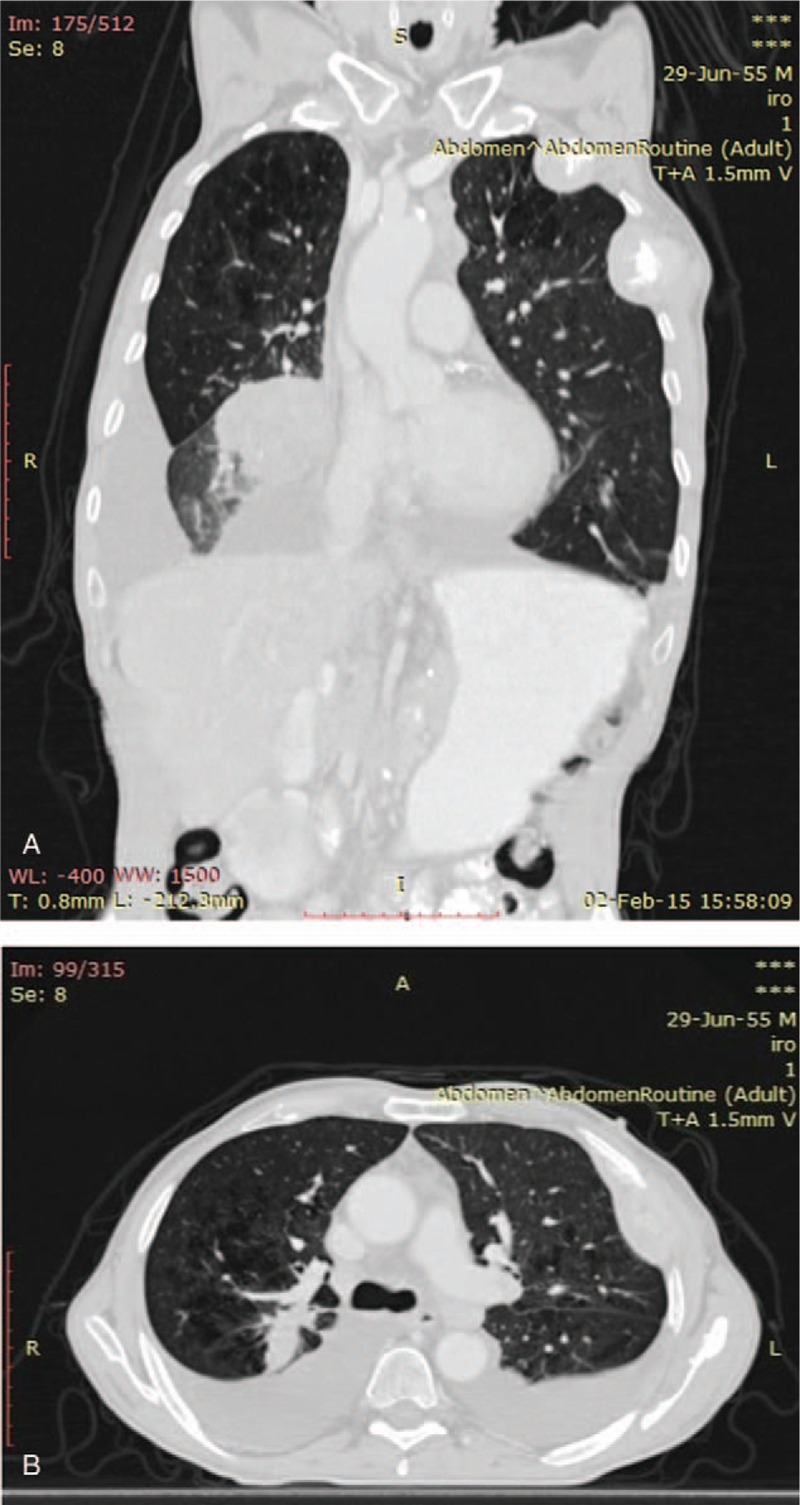
CT scan of the lungs showing progressive disease.

In March 2013, the patient was admitted at the Medical Oncology Clinic. His performance status upon admission was 2 on the ECOG scale. He underwent a first-line chemotherapy protocol with cisplatin (75 mg/m^2^) and gemcitabine (1000 mg/m^2^) on days 1 and 8 every 3 weeks. The patient did not benefit from EGFR and ALK testing since these were not available in our country at the time. Six cycles were administered and well tolerated clinically, the main side-effect being grade 1 vomiting. The first imagistic assessment in June 2013 showed a partial response to treatment according to RECIST 1.1., and a subsequent evaluation performed in December 2013 showed stable disease. After 6 more months, in June 2014, the CT scan of the thorax and abdomen described progressive disease into the lungs and bones. A second-line palliative chemotherapy protocol was initiated using 75 mg/m^2^ of Docetaxel every 3 weeks. The imagistic assessment after 6 cycles showed progressive disease, which lead to the decision to switch treatment to Erlotinib in October 2014, despite the fact that the patient had not presented EGFR mutation. At that time in our country erlotinib was reimbursed for patients with stage IV lung adenocarcinoma without EGFR mutations after first-line chemotherapy. There were no side-effects to erlotinib. In February 2015, the patient had progressive disease and developed pleural effusion (Fig. 4) and after 3 months he died.

## 3 Discussion

Metastasizing is the biological phenomenon accounting for 90% of deaths in cancer patients. Secondary lesions may be located in different organs depending on the particular specificity of the tumor cells in relation to that organ and on the vascularization distribution.^[9]^ The bone is the third most frequent site for solid tumor metastases, and the spine, ribs, and pelvis are most often affected. Acrometastases are very rare, as they occur in only 0.1% of the patients diagnosed with bone metastases. Handley was the first to report such a rare manifestation in the hand in 1906.^[5,10]^ Metastases in the hand are infrequent, and as a sign of cancer onset they are extremely rare.^[10]^ According to Flynn's review, the most commonly involved finger is the middle one, followed by the thumb. The distal phalanx is the part most frequently affected, due to the reduced blood flow. Metastases are least likely in the carpal area.^[7]^ Although most data on acrometastases is incomplete and based on retrospective studies, it has been possible to conclude that acrometastases are more common in men than in women, and they mostly affect people between 40 and 80 years old. In a study on 57 British patients with acrometastases, the average age at diagnosis was 63.1. The most common primary tumors accounting for the acrometastases were lung tumors, followed by kidney, and breast tumors. The fact that men are more frequently affected may be explained by the strong correlation between smoking and lung cancer.^[8,11]^ The mechanism underpinning this biological phenomenon is yet to be fully understood, although several hypothesis have been put forward.

Seed and soil theory states that the bone is a fertile ground for the growth of metastatic tumor cells. Once they enter the system, the tumor cells travel through the bone marrow and then migrate through sinusoids toward the surface of the bone. Another explanation is that the bones of distal extremities contain less red marrow than other bones and are at greater distance from the primary tumor. To overcome these unfavorable conditions, several factors are needed in order to facilitate the migration, adhesion, and invasion of the tumor cells.^[12]^ Increased blood flow is considered one such factor favoring the occurrence of acrometastases. This hypothesis is supported by the fact that acrometastases are more common in the dominant hand, which has greater blood flow and is more exposed to trauma. Microscopically, it is presumed that trauma decreases the resistance of support tissues and therefore allows tumor emboli to settle in the skeletal tissue. Other authors suggest that chemotactic factors like prostaglandins released following trauma could be responsible for cellular migration and adherence to the bone tissue.^[5,8]^

The symptoms caused by acrometastases are nonspecific, which is why acrometastases can be easily mistaken for other disorders such as arthritis, osteomyelitis, trauma, gout, Paget disease, cysts, benign, or malignant tumors of the bones or skin.^[7]^ Omission of the acrometastases diagnosis can also be due to the hands not being included in standard CT scanning areas.^[2]^ The key symptom which may direct the physician to suspect neoplasm is pain that does not improve to movement or to NSAIDs and opioids.^[13]^

Because acrometastases are signs of disseminated disease, in the literature they are generally associated with poor prognosis. Thus, the average survival of such patients has been reported as 6 to 7 months only.^[2,6,8]^ Despite therapeutic progress, the overall survival has not been improved significantly in the last 25 years.^[7]^ In previous studies, there were no statistically significant differences between the survival of patients with acrometastases depending on the location and number of the metastases or the histopathological nature of the primary tumor.^[4]^ However, more recent data is unavailable, so at present it is possible that the median overall survival is actually higher.^[14]^ Our patient had a good survival. He lived for 25 months from the time of the diagnosis, significantly longer than other patient data reported in the literature. This may be due to the lack of updated information, but also to the fact that, in some cases, acrometastases might not be a reliable indicator of poor prognosis. Another aspect that could explain the patients’ longer survival is the individual biology of tumors which determine acrometastasis about which there is little known information.

In the treatment of acrometastases, there is no well established standard.^[11]^ Surgery, radiotherapy and antalgics play an important role. Healey et al attempted an assessment of treatment methods in a study of 29 patients. They concluded that amputation is the best therapeutic approach for patients with chances of survival beyond 5 months.^[7]^ Also, it is considerably beneficial for cases with refractory pain – a common occurrence in cancer patients^[15]^ or intolerable side-effects to opioids.^[7,16]^ However, other authors have reported good results from more conservatory treatments such as radiotherapy and pain therapy due to unfavorable prognosis. Radiotherapy has the advantage of being able to alleviate pain and partially restore function in the fingers and hand, especially in advanced cases in which amputation is not possible or if the patient does not consent to it. Undoubtedly, apart from the local treatment of acrometastases, mention should be made that the primary neoplasm requires systemic treatment and in most cases that includes chemotherapy, targeted molecular therapies, or immunotherapy.^[8,17]^ The treatment of choice in the presented case was initial amputation of the distal phalanx because the patient was in severe pain not responding to the usual medication.

## 4 Conclusions

Acrometastases are extremely rare and may be the first sign of lung cancer. Physicians from non-oncological medical specialties who encounter patients with tumors located in the hand may experience difficulties in establishing prompt and complete diagnosis, while early diagnosis is crucial if good results are to be obtained in such cases. Although most patients with acrometastases have a poor survival in some cases the survival can be much longer questioning the role of cancer biology.

## Author contributions

**Funding acquisition:** Mihai Vasile Marinca, Bogdan Gafton, Dana Elena Clement.

**Investigation:** Iuliana Hunea Zamisnicu.

**Resources:** Mihai Vasile Marinca, Bogdan Gafton, Dana Elena Clement, Irina Demsa.

**Software:** Anca Maria Adavidoaiei.

**Supervision:** Mihai Vasile Marinca, Bogdan Gafton, Dana Elena Clement, Lucian Miron.

**Writing – original draft:** Vlad-Adrian Afrăsânie, Ionut Gabriel Funingănă, Marius-Ionut Păduraru.

**Writing – review & editing:** Vlad-Adrian Afrăsânie, Teodora Alexa-Stratulat.
